# Social and clinical predictors of short- and long-term readmission after a severe exacerbation of copd

**DOI:** 10.1371/journal.pone.0229257

**Published:** 2020-02-27

**Authors:** Sara Fernández-García, Cristina Represas-Represas, Alberto Ruano-Raviña, Cecilia Mouronte-Roibás, Maribel Botana-Rial, Cristina Ramos-Hernández, Alberto Fernández-Villar

**Affiliations:** 1 Department of Pneumonology, University Hospital Complex of Vigo, Pontevedra, Spain; 2 Neumo Vigo I +i. Institute of Health Research Galicia Sur (IISGS, *Instituto de Investigación Sanitaria Galicia Sur*), Vigo, Pontevedra, Spain; 3 Department of Preventive Medicine and Public Health, University of Santiago de Compostela, Santiago de Compostela, Spain; 4 Consortium for Biomedical Research in Epidemiology and Public Health (CIBERESP, *Consorcio de Investigación Biomédica en Red de Epidemiología y Salud Pública)*, Madrid, Spain; University of Torino, ITALY

## Abstract

**Introduction and objectives:**

The aim of this study was to evaluate the predictive ability of multiple social, and clinical factors for readmission after a severe acute exacerbation of COPD (AECOPD) during various time periods.

**Methods:**

We performed a prospective cohort study in which recruited patients with AECOPD. We systematically collected numerous clinical (symptoms, pulmonary function, comorbidities, and treatment) and social (financial situation, housing situation, family support, caregiver overload, ability to perform activities, and risk of social exclusion) variables using several questionnaires and indices. The patients were followed closely for one year and readmissions at 30, 60, and 365 days were analysed.

**Results:**

253 patients were included, aged 68.9±9.8years, FEV_1_ = 42.1%±14.2%, and a Charlson’s index = 1.8±0.9. Of these patients, 20.2%, 39.6%, and 63.7% were readmitted within the first 30, 90, and 365 days after discharge, respectively. In the multivariate model applied, the variables that were independently associated with readmission over all three periods of the analysis were dependence to perform basic activities of daily living (BADLs) (odds ratio [OR] = 2.10–4.10) and a history of two or more admissions within the previous year (OR = 2.78–3.78). At 90 days, a history of bacterial isolates in a previous sputum culture (OR = 2.39) and at 365 days, a high grade of dyspnoea (OR = 2.51) and obesity (OR = 2.38) were also identified as predictors of hospital readmission.

**Conclusions:**

The patients’ limitation to perform BADLs and their history of admissions for AECOPD were the best predictive variables for the likelihood of readmission when adjusted for many other social and clinical variables, regardless of the time period considered for such prediction.

## Introduction

Chronic obstructive pulmonary disease (COPD) is one of the most frequent respiratory diseases of our time, with high associated morbidity and mortality rates, and an important social and economic burden that is expected to increase in the coming years [1,2]. This condition is characterized by its chronicity and frequent acute exacerbations that contribute to a significant deterioration of the patients’ health, affect the disease’s progression and control, and result in a strong demand for health care resources, with the consequent socioeconomic impact, which accounts for 60%-70% of the costs of this disease [1–3]. An additional problem associated with this condition is that a significant number of patients admitted to the hospital for an acute exacerbation of chronic obstructive pulmonary disease (AECOPD) will be readmitted in the following weeks or months, as demonstrated in many studies reporting readmission rates of 20%, 35%, and 60% at 30, 90, and 365 days, respectively [3–10]. The detection of factors that may allow us to predict these events has been the subject of multiple research studies carried out in recent years, as this would allow us to stratify the risks more accurately and apply interventions aimed at the most vulnerable groups of patients [3–13].

Although the available literature is very heterogeneous, the most frequently described predictive variables can be categorized as those related to the patient (previous admissions, severity of the condition, need for oxygen therapy or at-home ventilation, older age, low quality of life, comorbidities, low socioeconomic level, low therapeutic adherence, or active smoking) and those related to the health system (duration of previous hospital admissions, absence of a defined follow-up program, or poor health education) [3–13]. Although some studies have reported that the patients’ deficient social situation are predictive factors for readmission and mortality, the analyses carried out to date have been very scarce and solely based on data regarding the patients’ level of education, economic income, or the characteristics of the patients’ home or caregivers and which have not been collected systematically [14–17]. Experts in this field state that these variables must be included in the predictive scales [17]. In fact, the current US program that financially penalizes hospitals with a great number of readmissions has been criticized due to the fact that it does not make any adjustments for social aspects [18,19]. Thus, it seems surprising that little emphasis has been placed on social dysfunction (financial situation, housing situation, family support, caregiver overload, ability to perform basic and instrumental activities, and risk of social exclusion), as the benefit that can be obtained by improving these variables would have a greater impact on the patient than small improvements achieved in their overall situation and pulmonary function [14–17].

Moreover, most studies have only analysed these factors at 30 or 90 days of hospital discharge, and the long-term evidence is much more limited, with no references on whether these predictive factors vary over time until the patients’ readmission [4–11].

In this study we analysed the ability of multiple social, clinical, and demographic variables to predict the likelihood of a new readmission after a severe AECOPD from a formal point of view and during various time periods.

## Methods

### Design and setting

We designed a prospective cohort study in which we consecutively recruited patients with an index admission (first admission during the study period) due to a primary diagnosis of AECOPD at the Pneumology Service of a tertiary public hospital with a reference population of 375,000 inhabitants. The patients were recruited over a period of one year (2 January 2017 to 31 December 2017).

Patients who refused to participate and those in whom the diagnosis of COPD or AECOPD was ruled out during admission or follow-up were excluded from the study [1,2].

### Data collection

The material and methods used in this study were explained in more detail in a previous publication [20]. On the 3^rd^ and 4^th^ day of admission, depending on the patient's clinical situation, the health personnel and a social worker requested the patients and caregiver a written and informed consent *f*or their inclusion in the study and systematically collected their social, demographic, and clinical information by reviewing their electronic medical records and interviewing both the patients and their caregivers. The patients’ demographic information, level of education, place of residence, usual means of transport, monthly income, and type of pension (contributory or not) were registered. In addition, their employment regime; type of housing (flat, house, nursing home, hotel); living characteristics (living alone); caregiver availability and remuneration; previous contacts with social services; dependence to perform basic activities of daily living (BADLs) measured according to Barthel’s index [21] and to perform instrumental activities measured according to Lawton and Brody’s index [22] prior to the admission; score in Barber’s social fragility questionnaire [23]*; social or family situation measured according to Gijón’s socio-familial evaluation scale [24]; and the informal caregivers’ overload (when applicable) measured according to Zarit’s test [25]. The social variables were transformed into dichotomous variables based on the recommended cut-off points [21,22,24,25].

The patients’ clinical variables, month of admission, body mass index, previous and current smoking history, non-smoking causes of the COPD, average daily consumption of alcohol and other drugs, previous vaccinations (flu and pneumococcal vaccines), hospital admissions due to an AECOPD, cultures of respiratory samples performed within the previous year, impact of the disease and degree of dyspnoea prior to the AECOPD assessed with the COPD Assessment Test (CAT) [26] and the modified scale of the Medical Research Council (mMRC) [27], FEV_1_ value in the last spirometry performed, and levels of eosinophils and existence of evidence of anaemia in the blood tests performed at admission were all collected. The coexistence of other comorbidities was also recorded using Charlson’s Comorbidity Index [28] and Goldberg’s Anxiety and Depression Scale [29], as well as the presence of obstructive sleep apnoea syndrome or atrial fibrillation. These were all grouped into a single variable for all cardiovascular diseases included in Charlson’s index to which atrial fibrillation was added. In addition, the GOLD guidelines [1] were used to classify the patients according to their FEV_1_. Other dichotomous variables described in the results section were also created with the values of these indices and questionnaires (mMRC, CAT, Charlson, Goldberg).

At discharge, the mean stays, the types of bronchodilator drugs administered (with or without inhaled corticosteroids), and the need for oxygen therapy or at-home non-invasive ventilation were also recorded.

All patients were followed for one year after their initial admission by reviewing their electronic medical records at 30, 90, and 365 days. The end of the follow-up was set at 365 days of their index admission or at the time of death (when applicable). The patients or their relatives were contacted by telephone in those cases in which there were any doubts or lack of information. All non-scheduled hospital admissions due to a medical pathology (non-surgical or traumatological) within the 365 days following the patients’ discharge from their index admission was considered a readmission. In those cases in which the patients had been readmitted on several occasions, only the first one following their discharge from the index admission was included.

The STROBE guidelines [30] were followed for the methodology and the notification of results.

The sample size was calculated based on an estimated percentage of readmissions at three months of 35% and a confidence level of 95%, which allowed us to work with a statistical power of 97% to detect medium effect sizes and of 70% for small effect sizes of the predictive variables. The statistical power was higher than 80% as of 200 subjects and higher than 85% with 250 subjects.

The study was approved by the Research Ethics Committee of Galicia (code 2016/524).

### Statistical analysis

An analysis was performed describing the number of cases and their percentage in the case of the qualitative variables, and the 95% confidence interval (95% CI), the arithmetic mean, and the standard deviation in the case of the quantitative ones. Contingency tables were created for the categorical variables using the number of cases and their percentage. These were subsequently compared using the chi-squared test or Fisher’s exact test. The comparison of quantitative variables was performed using Student's T test. In order to identify the independent predictors of readmission over the three time periods analysed, a conditional logistic regression analysis was carried out in which all variables with a *p* <0.10 in the univariate model were included. The odds ratio (OR) was calculated with its 95% confidence interval (95%CI). Except for the age and the pack-years index, all other numerical variables included in the model were transformed into a dichotomous expression. The analysis was carried out using statistical software package IBM SPSS Statistics 21 (IBM Corporation, Armonk, NY, USA).

## Results

**Fig 1:**
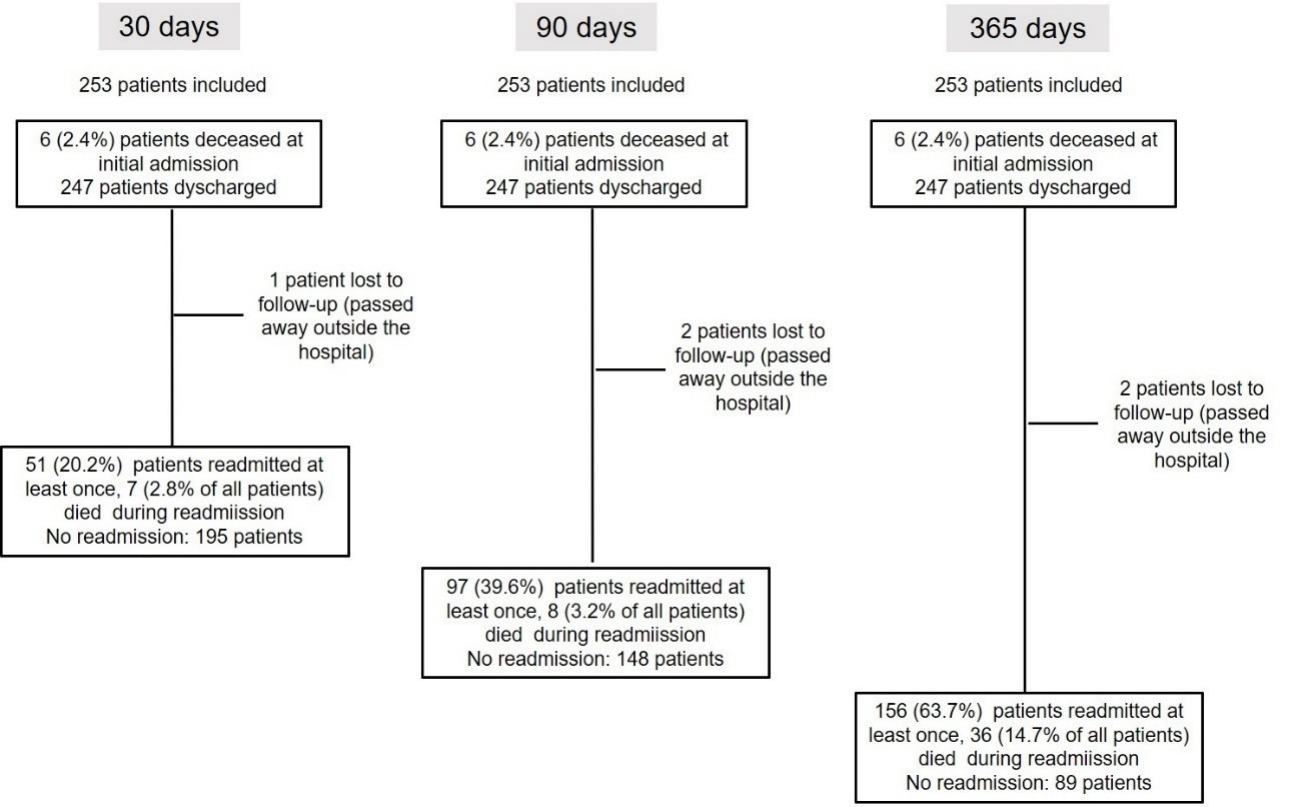
Flowchart showing the events that took place over the three study periods.

A total of 253 patients were included in the study. Six of the patients (2.4%, 95%CI:0.5%-4%) who had agreed to participate in the study passed away during the initial admission process, three did not agree to participate in the study, and two were not included due to their severe condition. Fig 1 shows a flowchart describing the events that took place over the three time periods analysed. Tables 1 and 2 include a description of the global sample and the univariate analysis of the social, demographic, and clinical variables over the three time periods analysed.

**Table 1:** Univariate analysis of the social and demographic variables predictive of readmissions at 30, 90, and 365 days.

Social variables	Total (253)	30 days (246)			90 days (245)			365 days (245)		
		Readmitted N = 51	Not readmitted N = 195	*p*	Readmitted N = 97	Not readmitted N = 148	*p*	Readmitted N = 156	Not readmitted N = 89	*p*
Male sex (%)	195 (77.5)	44 (86.3)	144 (73.8)	0.07	79 (81.4)	109 (73.6)	0.15	124 (79.5)	64 (71.9)	0.18
Age (years)^1^	68.9 ± 9.8	69.5 ± 9.8	68.3 ± 9.7	0.43	69.5 ± 9.5	67.9 ± 9.7	0.21	69.8 ± 9.5	66.5 ± 9.6	0.01
Level of education (%)	214 (84.6)	44 (86.3)	166 (85.1)	0.84	85 (87.6)	124 (83.8)	0.40	138 (88.5)	71 (79.8)	0.07
Rural area of residence (%)	136 (54.0)	24 (47.1)	109 (56.2)	0.24	49 (50.5)	83 (56.5)	0.36	77 (49.7)	55 (61.8)	0.06
Income <800 € (%)	136 (55.5)	28 (56.0)	108 (57.4)	0.85	53 (56.4)	83 (57.6)	0.84	89 (58.6)	47 (54.7)	0.55
No income, pension or non-contributory pay (%)	8 (3.2)	1 (2.0)	7 (3.6)	0.48	2 (2.1)	5 (3.4)	0.70	4 (2.6)	3 (3.4)	0.71
Actively employed (%)	16 (6.3)	2 (3.9)	14 (7.2)	0.31	4 (4.1)	12 (8.1)	0.21	6 (3.8)	10 (11.2)	0.02
Distance to the hospital >20 km (%)	32 (12.7)	5 (9.8)	25 (12.9)	0.39	9 (9.3)	21 (14.3)	0.46	17 (11.0)	13 (14.6)	0.69
Use of a personal means of transport (%)	89 (35.2)	13 (25.5)	75 (38.5)	0.08	28 (28.9)	60 (40.5)	0.06	47 (30.1)	41 (46.1)	0.01
Use of social services resources (%)	58 (22.9)	13 (25.5)	41 (21.0)	0.49	23 (23.7)	31 (20.9)	0.61	37 (23.7)	17 (19.1)	0.40
Living alone (%)	54 (21.4)	7 (13.7)	44 (22.7)	0.16	15 (15.6)	36 (24.3)	0.10	32 (20.6)	19 (21.3)	0.89
With a caregiver (%)	117 (49)	28 (54.9)	85 (43.6)	0.15	55 (56.7)	57 (38.5)	0.005	78 (50.0)	34 (38.2)	0.07
With a caregiver (unpaid) (%)^1^	99 (83.2)	26 (92.9)	68 (80.0)	0.11	48 (87.3)	45 (78.9)	0.24	68 (87.2)	25 (73.5)	0.07
Zarit (caregiver overload) ^1^	51.4 ± 14.2	52.8 ± 14.7	49.8 ± 13.7	0.37	52.9 ± 15.7	48.3 ± 11.8	0.14	52.1 ± 14.0	46.8 ± 13.6	0.12
Any overload (Zarit test) (%)^1^	63 (69.2)	17 (77.3)	41 (64.1)	0.25	32 (74.4)	26 (61.9)	0.21	46 (75.4)	12 (50.0)	0.02
Barthel’s questionnaire^1^	88.3 ± 17.4	81.1 ± 21.8	90.7 ± 15.5	0.004	84.7 ± 19.1	91.6 ± 15.5	0.003	85.7 ± 19.1	94.4 ± 12.0	0.0001
Any degree of dependence (Barthel’s questionnaire) (%)	127 (46.2)	34 (66.7)	77 (39.5)	0.001	58 (59.8)	52 (35.1)	0.0001	88 (56.4)	22 (24.7)	0.0001
Dependence (Barber’s questionnaire) (%)	226 (89.3)	48 (94.1)	171 (87.7)	0.19	93 (95.9)	125 (84.5)	0.005	145 (92.9)	73 (82.0)	0.009
Lawton & Brody’s questionnaire^1^	4.8 ± 2.5	3.9 ± 2.3	5.1 ± 2.5	0.001	4.2 ± 2.4	5.2 ± 2.4	0.001	4.3 ± 2.4	5.7 ± 2.3	0.0001
Dependence (Lawton & Brody’s questionnaire) (%)	200 (79.1)	46 (90.2)	148 (75.9)	0.02	86 (88.7)	107 (72.3)	0.002	133 (85.3)	60 (67.4)	0.001
Gijón’s socio-familial questionnaire^3^	10.8 ± 3.1	10.6 ± 2.7	10.8 ± 3.2	0.56	10.7 ± 2.9	10.9 ± 3.1	0.54	11.1 ± 3.1	10.3 ± 3.0	0.08
Risk/problem according to Gijón’s socio-familial questionnaire (%)	162 (64)	31 (60.8)	126 (64.6)	0.61	61 (62.9)	95 (64.2)	0.83	106 (67.9)	50 (56.2)	0.06

^1^Calculated in relation to those with a caregiver.

^2^Calculated with respect to patients with an unpaid caregiver.

^3^Expressed as a mean ± standard deviation.

**Table 2:** Univariate analysis of the demographic and clinical variables with respect to the readmissions at 30, 90, and 365 days.

Clinical variables	Total (253)	30 days (246)			90 days (245)			365 days (245)		
		Readmitted N = 51	Not readmitted N = 195	*p*	Readmitted N = 97	Not readmitted N = 148	*p*	Readmitted N = 156	Not readmitted N = 89	*p*
BMI (kg/m^2^)^1^	27.5 ± 6.4	26.7 ± 6.1	27.7 ± 6.5	0.30	26.9 ± 6.3	27.7 ± 6.3	0.30	27.8 ± 6.8	26.6 ± 5.3	0.13
BMI >30 kg/m^2^ (%)	82 (32.5)	16 (32.0)	61 (31.8)	0.97	32 (33.7)	44 (30.1)	0.56	56 (36.4)	20 (23.0)	0.03
Active smoker (%)	96 (37.9)	15 (29.4)	80 (41.0)	0.13	31 (32.0)	63 (42.6)	0.09	55 (35.3)	39 (43.8)	0.18
Pack-years index^2^	54.2± 29.1	39.9 ± 27.5	53.2 ± 36.1	0.28	56.4 ± 35.3	53.7 ± 33.9	0.59	58.5 ± 36.4	48.2 ± 29.6	0.04
Strict non-smokers (%)	9 (3.6)	2 (3.9)	7 (3.6)	0.91	3 (3.1)	6 (4.1)	0.49	6 (3.8)	3 (3.4)	0.57
High alcohol consumption (%)	52 (20.6)	9 (17.6)	42 (21.5)	0.54	16 (16.5)	35 (23.6)	0.17	34 (21.8)	17 (19.1)	0.61
Drug abuse (%)	21 (8.4)	2 (3.9)	5 (2.6)	0.32	3 (3.1)	4 (2.7)	0.69	4 (2.6)	3 (3.4)	0.93
Admissions within the previous year^1^	0.8 ± 1.1	1.1 ± 1.3	0.7 ± 1.1	0.04	1.2 ± 1.3	0.5 ± 0.9	0.0001	1.0 ± 1.3	0.4 ± 0.7	0.0001
≥2 admissions within the previous year (%)	51 (20.2)	18 (35.3)	31 (15.9)	0.002	35 (36.1)	14 (9.5)	0.0001	41 (25.3)	8 (9.0)	0.001
Positive sputum (%) culture within the previous year	65 (25.8)	18 (35.3)	46 (23.7)	0.09	37 (38.1)	27 (18.4)	0.001	48 (30.8)	16 (18.2)	0.03
Flu vaccination (%)	211 (83.4)	46 (90.2)	158 (81.0)	0.12	83 (85.6)	120 (81.1)	0.36	131 (84.0)	72 (80.9)	0.53
Pneumococcal vaccination (%)	138 (54.5)	33 (62.3)	102 (52.3)	0.18	56 (57.7)	77 (52.0)	0.38	88 (56.4)	45 (50.6)	0.37
Eosinophil blood count (%)^1^	0.9 ± 1.1	0.9 ± 1.1	0.9 ± 1.2	0.81	0.9 ± 1.1	0.9 ± 1.2	0.84	0.9 ± 1.1	0.9 ± 1.2	0.95
CAT score^1^	19 ± 7.3	19.7 ± 6.7	18.6 ± 7.4	0.34	20.3 ± 6.7	17.8 ± 7.4	0.01	19.6 ± 6.7	17.2 ± 7.7	0.01
CAT score>10 (%)	220 (87.0)	46 (90.2)	167 (85.6)	0.39	89 (91.8)	123 (83.1)	0.05	142 (91.0)	70 (79.7)	0.006
Dyspnoea (mMRC)^1^	2.2 ± 0.8	2.4 ± 0.7	2.1 ± 0.8	0.02	2.4 ± 0.7	2.1 ± 0.8	0.0001	2.4 ± 0.7	1.9 ± 0.7	0.0001
Dyspnoea (mMRC 3–4) (%)	94 (37.2)	47 (92.2)	158 (81.0)	0.05	89 (91.8)	115 (77.7)	0.004	140 (89.7)	64 (71.9)	0.0001
FEV_1_ value (% reference)^1^	42.1 ± 14.2	41.5 ± 14.3	42.4 ± 14.2	0.68	39.3 ± 14.4	44.2 ± 13.7	0.01	40.8 ± 14.7	44.8 ± 12.8	0.03
FEV_1_ value (ml)^1^	1132.5 ± 466.2	1175.5 ± 479.3	1127.4 ± 462.9	0.51	1098.5 ± 475.7	1166.2 ± 458.8	0.27	1093.6 ± 468.3	1221.3 ± 452.5	0.04
FEV_1_ value 50% reference (GOLD III-IV) (%)	177 (71.4)	36 (70.6)	136 (71.2)	0.93	73 (76.8)	98 (67.1)	0.10	113 (73.4)	58 (66.7)	0.27
Charlson’s index^1^	1.8 ± 0.9	2.1 ± 1.0	1.6 ± 0.8	0.003	1.8 ± 0.9	1.7 ± 0.9	0.23	1.8 ± 0.9	1.6 ± 0.9	0.10
Charlson’s index (≥ 2) (%)	132 (52.2)	33 (64.7)	94 (48.2)	0.03	54 (55.7)	73 (49.3)	0.33	88 (56.4)	39 (43.8)	0.05
Charlson’s index adjusted by age^1^	4.2 ± 1.5	4.6 ± 1.6	4.0 ± 1.3	0.01	4.4 ± 1.4	4.1 ± 1.4	0.10	4.3 ± 1.4	3.8 ± 1.4	0.004
Cardiovascular disease (%)	90 (35.6)	24 (47.1)	59 (30.3)	0.02	42 (43.3)	41 (27.7)	0.01	62 (39.7)	21 (23.6)	0.01
Diabetes mellitus (%)	57 (22.5)	13 (25.5)	43 (22.1)	0.60	18 (18.6)	38 (25.7)	0.19	36 (23.1)	20 (22.5)	0.91
Anaemia (%)	65 (25.7)	16 (31.4)	44 (22.6)	0.19	28 (28.9)	32 (21.6)	0.19	42 (26.9)	18 (20.2)	0.24
OSAS (%)	51 (20.2)	13 (25.5)	37 (19.0)	0.30	23 (23.7)	27 (18.2)	0.29	33 (21.2)	17 (19.1)	0.70
Goldberg’s questionnaire (total) ^1^	5.8 ± 3.9	6.3 ± 4.1	5.4 ± 3.8	0.17	6.0 ± 3.8	5.4 ± 3.8	0.18	5.9 ± 3.8	5.1 ± 3.9	0.12
Anxiety (Goldberg’s questionnaire) (%)	112 (44.4)	26 (52.0)	83 (42.6)	0.23	46 (47.9)	62 (41.9)	0.35	72 (46.5)	36 (40.4)	0.36
Depression (Goldberg’s questionnaire) (%)	131 (53.2)	25 (52.0)	102 (52.3)	0.96	53 (55.2)	75 (50.7)	0.49	88 (56.8)	40 (44.9)	0.07
Mean hospital stay^1^	8.1 ± 8.1	7.3 ± 4.8	7.7 ± 6.4	0.70	7.9 ± 4.8	7.4 ± 6.8	0.58	8.4 ± 6.9	6.3 ± 4.0	0.003
Mean hospital stay ≥7 days (%)	125 (49.4)	23 (45.1)	98 (50.3)	0.51	52 (53.6)	68 (45.9)	0.24	85 (54.5)	35 (39.3)	0.02
LABA at discharge (%)	201 (81.7)	44 (86.3)	181 (93.8)	0.08	84 (87.5)	140 (95.2)	0.03	140 (90.3)	84 (95.5)	0.95
LAMA at discharge (%)	227 (92.3)	43 (84.3)	157 (81.3)	0.62	76 (79.2)	123 (83.7)	0.37	123 (79.4)	76 (86.4)	0.17
IC at discharge (%)	153 (60.2)	33 (64.7)	119 (61.7)	0.69	62 (64.6)	89 (60.5)	0.52	105 (67.7)	46 (52.3)	0.02
At-home oxygen therapy (%)	100 (39.5)	19 (37.3)	79 (40.9)	0.63	48 (50.0)	50 (30.4)	0.01	72 (46.5)	26 (29.5)	0.01
At-home non-invasive ventilation (%)	42 (16.7)	8 (15.7)	33 (16.9)	0.63	16 (16.5)	24 (16.2)	0.95	22 (14.1)	18 (20.2)	0.21

^1^Expressed as a mean ± standard deviation.

^2^Calculed in smokers and former smokers.

^3^Causes: alpha-1-antitrypsin deficiency (one man and two women), occupational (four men and two women), and biomass inhalation (two women).

Abbreviations: CAT: COPD Assessment Test; IC: inhaled corticosteroids; FEV_1_: forced expiratory volume in the first second; BMI: body mass index; mMRC: modified scale of the Medical Research Council; LABA: long-acting β2 agonist; LAMA: long-acting muscarinic receptor antagonists; OSAS: obstructive sleep apnoea syndrome.

One third (75%) of the participants were men aged 60–70 years old, with a low level of education, who generally lived in their own family homes located near the hospital, half of which were located in the city, and a significant number of whom did not have their own means of transport. Four patients lived in hotels (1.6%) and two (0.8%) stayed at nursing homes. Most of them were pensioners, and more than half had low incomes. A quarter of the patients lived or slept alone. Almost half had some degree of dependence to perform certain BADLs (70% had a moderate-severe dependence), the majority for instrumental activities, and 60% had a risk or socio-familial problems. Despite the above, only half of the participants had caregivers, almost all of whom were informal and mostly women (spouses or relatives) who frequently suffered some degree of overload. Notwithstanding the above, only one fifth of the patients had contacted social services.

Compared to patients who have caregivers, those who live alone were less dependent on basic activities (17.3% vs. 53.1%; p = 0.0001) and instrumental (55.8% vs. 85.1%; p = 0.0001).

Most of the patients were overweight or obese, with a functionally severe COPD and a grade 2–3 dyspnoea according to the mMRC. In almost all cases, the COPD was a result of tobacco consumption. More than two thirds of the patients continued to smoke daily and one fifth of them drank alcohol excessively. Of the less than 10% of the participants who were drug users, most were included in methadone programs and only six consumed other substances of abuse (heroin) on a frequent basis. Although Charlson’s index was low, slightly more than half of the patients had cardiovascular comorbidities, half of them suffered from anxiety or depression, and between one fourth and one fifth of them had diabetes, sleep apnoea, or mild anaemia. Forty per cent of the participants received at-home oxygen therapy and 15% received non-invasive ventilation.

Of all patients, 20.2% (95%CI: 16%-26%), 39.6% (95%CI: 33%-46%), and 63.7% (95%CI: 58%-70%) were first readmitted to the hospital within the first 30, 90, and 365 days after discharge, respectively. The average global rate of admissions was 1.3±1.6. Of the 156 patients who were readmitted, 83 (33.9%) were readmitted once and 73 (29.8%) were readmitted two or more times. Ninety-seven patients (62.2% of the total number of readmissions) were first readmitted within the first 90 days of discharge from their index admission.

All variables described in Tables 2 and 3 with a *p* <0.10 were included in the multivariate model, in such a way that if both the numerical variable and the dichotomous variable of the same predictor revealed an association, only the qualitative one was included. Table 3 outlines the variables that were independently associated with readmission over the three time periods analysed in the applied multivariate model, which, in all cases, were dependence to perform BADLs and a history of two or more admissions within the previous year. Table 4 shows the differences in the dependence of the readmitted and the non-readmitted patients according to the domains of Barthel’s index over the analysed time period. Significant differences were detected in all time periods with respect to activities related mainly to mobility, followed closely by personal care.

**Table 3:** Variables predicting readmission at 30, 90, and 365 days in the multivariate analysis.

Predictive variables	Readmissions at 30 days			Readmissions at 90 days			Readmissions at 365 days		
	OR	95% CI	*p*	OR	95% CI	*p*	OR	95% CI	*p*
Any degree of dependency (Barthel’s index)	2.77	1.43 - 5.37	0.003	2.10	1.13 - 3.85	0.018	4.10	2.15 - 7.79	0.001
≥2 admissions within the previous year	2.43	1.19 - 4.95	0.014	3.78	1.81 - 7.90	0.0001	2.78	1.15 - 6.78	0.023
≥1 positive sputum culture within the previous year	-	-	-	2.39	1.25 - 4.58	0.008	-	-	-
Grade 3–4 dyspnoea	-	-	-	-	-	-	2.51	1.18 - 3.35	0.017
Body mass index ≥30 kg/m^2^	-	-	-	-	-	-	2.38	1.21 - 4.66	0.018

**Table 4:** Comparison between the dependence of the readmitted and the non-readmitted patients based on Barthel’s index and classified according to the three time periods.

Any degree of dependence to perform activities included in Barthel’s index	Total (253)	30 days (246)			90 days (245)			365 days (245)		
		Readmitted N = 51	Not readmitted N = 195	*p*	Readmitted N = 97	Not readmitted N = 148	*p*	Readmitted N = 156	Not readmitted N = 148	*p*
Feeding (%)	33 (13.1)	14 (27.4)	17 (8.7)	0.001	18 (18.5)	13 (8.8)	0.06	26 (16.7)	5 (5.6)	0.02
Bathing (%)	73 (28.9)	18 (35.3)	50 (25.6)	0.17	35 (36.1)	32 (21.6)	0.01	53 (34.0)	14 (15.7)	0.002
Dressing (%)	81 (32.0)	24 (47.1)	53 (27.2)	0.02	38 (39.2)	38 (25.7)	0.05	63 (39.1)	15 (16.8)	0.001
Personal hygiene (%)	35 (13.8)	9 (17.6)	25 (12.8)	0.37	19 (19.6)	14 (9.5)	0.02	28 (17.9)	5 (5.6)	0.007
Toilet use (%)	48 (18.6)	14 (27.4)	31 (15.9)	0.18	23 (23.7)	21 (14.2)	0.16	35 (22.4)	9 (10.1)	0.05
Defecating (%)	38 (15.1)	14 (27.4)	23 (11.8)	0.02	19 (19.6)	18 (12.1)	0.21	28 (19.1)	9 (10.1)	0.19
Urinating (%)	36 (14.3)	13 (25.4)	23 (11.8)	0.02	18 (18.5)	18 (12.2)	0.38	30 (19.6)	6 (6.7)	0.01
Walking (%)	54 (21.4)	19 (37.2)	31 (15.9)	0.003	27 (27.9)	22 (14.8)	0.05	40 (25.6)	9 (10.1)	0.004
Climbing and descending stairs (%)	70 (27.8)	20 (39.2)	44 (22.6)	0.008	32 (33.0)	31 (21.0)	0.04	52 (33.3)	11 (12.3)	0.001
Transfers (%)	37 (14.6)	11 (21.6)	23 (11.8)	0.05	20 (20.6)	13 (8.8)	0.01	29 (18.6)	4 (4.5)	0.002

## Discussion

This is the first published study that specifically and thoroughly analyses the influence of social (economic situation, housing situation, setting, family support, caregivers’ burden, dependence to perform basic and instrumental activities, and risk of social exclusion), clinical, and demographic variables on the likelihood of short- and long-term readmission following discharge from an index admission due to a severe COPD.

Only the patients’ dependence to perform BADLs measured by means of a questionnaire not specifically designed for patients with respiratory pathologies and previous history of admissions were identified as predictors over any of the analysed time periods. Thus far, the available references regarding this topic are scarce and heterogeneous, as they were obtained from very diverse studies (from small case series to large databases of insurance companies) and centres forming part of health systems with varying forms of health care services and very disparate patient populations [7,8,10,14,31]. In contrast to the findings of other studies [7,8,10], in our research, the patients’ economic income, a low level of education, or poor family support (all determining factors of social and family risk) were not predictors of readmission, although it is true that most of our patients received some sort of economic compensation, had a place to live, and there were practically no cases of serious social isolation. It should also be noted that the Spanish Health System is universally accessible and free of charge at all levels of care, an aspect that could have influenced these results [31].

In our study, when adjusted, the variables of living alone, sleeping alone, or not having a caregiver were not related to a risk of readmission, as the patients that met these characteristics were much more independent to perform BADLs. However, the opposite was true for the patients with anxiety and depression. Thus, it seems that being dependent to perform the most BADLs is the social factor most closely related to a likelihood of readmission and, although this was already demonstrated in other acute or chronic diseases [32], the evidence in this regard for COPD is very limited. Some recent reviews have analysed the term "frailty" as a syndrome that includes disability, together with physical and cognitive deterioration, as a predictor of readmission [7,8]. However, the references mentioned are based on studies performed with patients with other pathologies, such as heart failure, and not specific to patients with COPD [33]. Some validation studies of predictive scales of readmission or mortality indirectly included some partial analyses of the influence of the patients’ dependence to perform BADLs on the rate of readmission. Echevarría *et al*., in a validation study of the PEARL index to predict readmission or mortality, proved that by broadening the MRC dyspnoea scale by including a level at which the patient needs help to get dressed or go to the bathroom, the predictive ability of the index improves and that it is better than other multidimensional indexes that only include clinical variables [34]. A similar finding was described in a small Spanish study using the Edmonton Scale of frailty [35]. Other studies have also described poor physical activity as a predictor of readmission [36].

Therefore, from this point of view, COPD should be considered more in terms of a "function" than a "disease", and an assessment of this domain should be added to the available predictive scales. Moreover, coordinated work with the social services should be implemented to improve the impact of the patients’ dependence to perform BADLs so as to reduce the rate of readmissions based on the findings obtained in this respect for other pathologies [27]. This result could also reinforce the role of Pulmonary Rehabilitation in reducing short- and long-term readmission following discharge from an index admission due to a severe COPD [18].

The use of a non-specific index of respiratory pathology in this study, such as Barthel’s index [21], which is influenced not only by dyspnoea, but also by other functional mobility or cognitive impairments, or even by the patients’ specific degree of support, could have influenced the strength that this variable had in our study. It is likely that Barthel’s index reflects in a much more global way the true ability of each patient to perform their most basic functions [33, 37, 38]. Also, it could explain that other variables such as a low pulmonary function, a high number of comorbidities, or a high grade of dyspnoea, which in other studies were predictors of readmission, were only predictors in the unadjusted analysis in only one of the temporary evaluations carried out in our study. Although all of them are related to the patients’ degree of dependence to perform BADLs, this relationship is not very powerful [39].

A history of previous admissions due to COPD was also an independent predictor over the three time periods that were analysed in our study, especially in the case of patients who were readmitted two or more times within the previous year. This finding was also demonstrated in other studies [7,8] and, in fact, was included in several validated predictive indices such as the PEARL, BODEX, or CODEX indices, among others [33]. Although it could be considered more of a consequence than a cause, its independent predictive capacity leads us to believe that some patients may have a COPD with a particularly strong demand for hospital management, added to the current limitations of the definition of severe exacerbation and the hospitalization criteria, which are not exempt from a certain degree of subjectivity and vary greatly among hospitals [6,9].

Other variables such as a history of bacterial isolation in a sputum culture or a high grade of dyspnoea have also been identified as predictors of hospitalization at 90 and 365 days, respectively. The presence of bacteria in the respiratory tract contributes to increase and perpetuate the chronic inflammation inherent to the COPD and requires close monitoring of patients with this condition [9]. The degree of dyspnoea has also been used as a predictive variable in many other studies also analysing readmission rates [7,8]. The reason why dyspnoea was only an independent predictor in our study when analysing long-term readmissions could have been the number of long-term events and the complexity of the interaction among the great number of variables included in the study.

Obesity was also identified as a long-term predictor of readmission. However, the available evidence concerning this factor in cases of COPD is very controversial, given that, although some authors consider it to be a protective factor [39], more recent studies suggest the opposite [40].

The limitations of our study included, in the first place, the likelihood that the studied population and the care provided to the patients cannot be extrapolated to many other countries, especially with respect to the social variables. Nevertheless, the clinical and demographic characteristics analysed in our study were very similar to those described in an extensive European audit [6]. Another limitation was that we used scales that have not yet been widely validated in COPD to evaluate aspects such as the patients’ dependence to perform basic and instrumental activities, caregiver overload, or social risk. However, we believe that in this disease, in which a considerable number of patients have multiple comorbidities, indices such as Barthel’s index may reflect a more global picture of the patients’ situation than others that exclusively focus on dyspnoea as the main conditioning factor of their limitations [38]. However, it is possible that the use of a questionnaire that combines dependence for activities in relation to dyspnoea such as those proposed by other authors could also have its usefulness in predicting these events [41].

As for the strengths of our study, we would like to highlight the consecutive inclusion of patients, the systematic evaluation of many social variables by personnel specializing in social work, and the close monitoring carried out, which minimized the loss of information. Finally, we consider that the one-year follow-up was sufficiently long to analyse the impact of the patients’ characteristics on the rate of readmissions for AECOPD over time.

To conclude, the patients’ limitation to perform BADLs and their history of admissions for AECOPD within the previous year are the best predictive variables of the probability of readmission when adjusted for many other social and clinical variables, regardless of the time period considered for the prediction. Although additional studies are needed in this respect, we believe that the patients’ dependence to perform BADLs should be included in the validation of new predictive scales for readmission after severe exacerbations.

## References

[1] Global Strategy for the Diagnosis, Management, and Prevention of COPD. Global Initiative for Chronic Obstructive Pulmonary Disease (GOLD) 2019. Available in: www.goldcopd.org

[2] Working group of the GesEPOC. Clinical Practice Guideline for the Diagnosis and Treatment of Patients with Chronic Obstructive Pulmonary disease (COPD)–the Spanish COPD Guideline (GesEPOC). Arch Bronconeumol. 2017;53(Supl 1):1–64.

[3] Halpin DM, Miravitlles M, Metzdorf N, Celli B. Impact and prevention of severe exacerbations of COPD: a review of the evidence. Int J Chron Obstruct Pulmon Dis. 2017;12:2891–2908. 10.2147/COPD.S139470 29062228 PMC5638577

[4] Mulpuru S, McKay J, Ronksley PE, Thavorn K, Kobewka DM, Forster AJ. Factors contributing to high-cost hospital care for patients with COPD. Int J Chron Obstruct Pulmon Dis. 2017;12:989–995. 10.2147/COPD.S126607 28392683 PMC5373828

[5] Escarrabill J, Torrente E, Esquinas C, Hernández C, Monsó E, Freixas M, et al Clinical audit of patients hospitalized due to COPD exacerbation. MAG-1 Study. Arch Bronconeumol. 2015;51:483–9. 10.1016/j.arbres.2014.06.023 25447590

[6] Hartl S, Lopez-Campos JL, Pozo-Rodriguez F, Castro-Acosta A, Studnicka M, Kaiser B, et al Risk of death and readmission of hospital-admitted COPD exacerbations: European COPD Audit. Eur Respir J. 2016;47:113–21. 10.1183/13993003.01391-2014 26493806

[7] Bahadori K, FitzGerald JM. Risk factors of hospitalization and readmission of patients with COPD exacerbation—systematic review. Int J Chron Obstruct Pulmon Dis. 2007;2:241–51. 18229562 PMC2695199

[8] Shah T, Press VG, Huisingh-Scheetz M, White SR. COPD Readmissions: Addressing COPD in the Era of Value-based Health Care. Chest. 2016;150:916–926. 10.1016/j.chest.2016.05.002 27167208 PMC5812767

[9] Mantero M, Rogliani P, Di Pasquale M, Polverino E, Crisafulli E, Guerrero M, et al Acute exacerbations of COPD: risk factors for failure and relapse. Int J Chron Obstruct Pulmon Dis. 2017;12:2687–2693. 10.2147/COPD.S145253 28932112 PMC5598966

[10] Jacobs DM, Noyes K, Zhao J, Gibson W, Murphy TF, Sethi S, et al Early Hospital Readmissions after an Acute Exacerbation of Chronic Obstructive Pulmonary Disease in the Nationwide Readmissions Database. Ann Am Thorac Soc. 2018;15:837–845. 10.1513/AnnalsATS.201712-913OC 29611719 PMC6207114

[11] Lindenauer PK, Dharmarajan K, Qin L, Lin Z, Gershon AS, Krumholz HM. Risk Trajectories of Readmission and Death in the First Year after Hospitalization for Chronic Obstructive Pulmonary Disease. Am J Respir Crit Care Med. 2018;197:1009–1017. 10.1164/rccm.201709-1852OC 29206052 PMC5909167

[12] Raghavan D, Bartter T, Joshi M. How to reduce hospital readmissions in chronic obstructive pulmonary disease? Curr Opin Pulm Med. 2016;22:106–12. 10.1097/MCP.0000000000000245 26814142

[13] Press VG, Konetzka RT, White SR. Insights about the economic impact of chronic obstructive pulmonary disease readmissions post implementation of the hospital readmission reduction program. Curr Opin Pulm Med. 2018;24:138–146. 10.1097/MCP.0000000000000454 29210750 PMC5810972

[14] Wong AW, Gan WQ, Burns J, Sin DD, van Eeden SF. Acute exacerbation of chronic obstructive pulmonary disease: influence of social factors in determining length of hospital stay and readmission rates. Can Respir J. 2008;15:361–4. 10.1155/2008/569496 18949105 PMC2679571

[15] Owens JM, Garbe RA. Effect of enhanced psychosocial assessment on readmissions of patients with chronic obstructive pulmonary disease. Soc Work Health Care. 2015;54:234–51. 10.1080/00981389.2015.1005269 25760490

[16] Coventry PA, Gemmell I, Todd C. Psychosocial risk factors for hospital readmission in COPD patients on early discharge services: a cohort study. BMC Pulm Med. 2011;11:49 10.1186/1471-2466-11-49 22054636 PMC3217878

[17] Press VG. Is It Time to Move on from Identifying Risk Factors for 30-Day Chronic Obstructive Pulmonary Disease Readmission? A Call for Risk Prediction Tools. Ann Am Thorac Soc. 2018;15:801–3. 10.1513/AnnalsATS.201804-246ED 29957037

[18] Press VG, Au DH, Bourbeau J, Dransfield MT, Gershon AS, Krishnan JA, et al Reducing Chronic Obstructive Pulmonary Disease Hospital Readmissions. An Official American Thoracic Society Workshop Report. Ann Am Thorac Soc. 2019;16:161–170. 10.1513/AnnalsATS.201811-755WS 30707066 PMC6812156

[19] Chatterjee P, Werner RM. The hospital readmission reduction program and social risk. Health Serv Res. 2019;54:324–326. 10.1111/1475-6773.13131 30848490 PMC6407346

[20] Fernández-García S, Represas-Represas C, Ruano-Raviña A, Mosteiro Añón M, Mouronte Roibas C, Fernández-Villar A. Social Profile of Patients Admitted for COPD Exacerbations. A Gender Analysis. Arch Bronconeumol. 2019 (epub ahead of print). 10.1016/j.arbres.2019.03.009 30982691

[21] González N, Bilbao A, Forjaz MJ, Ayala A, Orive M, Garcia-Gutierrez S, et al OFF (Older Falls Fracture)-IRYSS group. Psychometric characteristics of the Spanish version of the Barthel Index. Aging Clin Exp Res. 2018;30:489–497. 10.1007/s40520-017-0809-5 28770477

[22] Vergara I, Bilbao A, Orive M, Garcia-Gutierrez S, Navarro G, Quintana JM. Validation of the Spanish version of the Lawton IADL Scale for its application in elderly people. Health Qual Life Outcomes. 2012;30;10:130 10.1186/1477-7525-10-130 23110491 PMC3541128

[23] Martín-Lesende I, Rodríguez-Andrés C. Utility of the Barber questionnaire to select individuals aged 75 years or more at risk of hospitalisation, institutionalisation or death. Rev Esp Geriatr Gerontol. 2005;40:335–44.

[24] Alarcón MT, González JL. The Gijon social-family scale, a useful instrument in the General Hospital. Rev Esp Geriatr Gerontol. 1998;33:178–9.

[25] Van Durme T, Macq J, Jeanmart C, Gobert M. Tools for measuring the impact of informal caregiving of the elderly: a literature review. Int J Nurs Stud. 2012;49:490–504. 10.1016/j.ijnurstu.2011.10.011 22078211

[26] Jones PW, Harding G, Berry P, Wiklund I, Chen W-H, Kline Leidy N. Development and first validation of the COPD Assessment Test. Eur Respir J. 2009;34:648–54. 10.1183/09031936.00102509 19720809

[27] Bestall J, Paul E, Garrod R, Garnham R, Jones P, Wedzicha J. Usefulness of the Medical research Council (MRC) dyspnea scale as a measure of disability in patients with chronic obstructive pulmonary disease. Thorax. 1999;54:581–6. 10.1136/thx.54.7.581 10377201 PMC1745516

[28] Charlson ME, Szatrowski TP, Peterson J, Gold J. Validation of a combined comorbidity index. J Clin Epidemiol. 1994;47:1245–51. 10.1016/0895-4356(94)90129-5 7722560

[29] Goldberg D, Bridges K, Duncan-Jones P, Grayson D. Detecting anxiety and depression in general medical settings. BMJ. 1988;297:897–9. 10.1136/bmj.297.6653.897 3140969 PMC1834427

[30] Vandenbroucke JP, von Elm E, Altman DG, Gøtzsche PC, Mulrow CD, Pocock SJ, et al STROBE Initiative. Strengthening the Reporting of Observational Studies in Epidemiology (STROBE): explanation and elaboration. Int J Surg. 2014;12:1500–24. 10.1016/j.ijsu.2014.07.014 25046751

[31] Pedersen MK, Meyer G, Uhrenfeldt L. Risk factors for acute care hospital readmission in older persons in Western countries: a systematic review. JBI Database System Rev Implement Rep. 2017;15:454–485 10.11124/JBISRIR-2016-003267 28178023

[32] Meddings J, Reichert H, Smith SN, Iwashyna TJ, Langa KM, Hofer TP, et al The Impact of Disability and Social Determinants of Health on Condition-Specific Readmissions beyond Medicare Risk Adjustments: A Cohort Study. J Gen Intern Med. 2017;32:71–80. 10.1007/s11606-016-3869-x 27848189 PMC5215164

[33] Aimo A, Barison A, Mammini C, Emdin M. The Barthel Index in elderly acute heart failure patients. Frailty matters. Int J Cardiol. 2018;254:240–241. 10.1016/j.ijcard.2017.11.010 29407098

[34] Echevarria C, Steer J, Heslop-Marshall K, Stenton SC, Hickey PM, Hughes R, et al The PEARL score predicts 90-day readmission or death after hospitalisation for acute exacerbation of COPD. Thorax. 2017;72:686–693. 10.1136/thoraxjnl-2016-209298 28235886 PMC5537524

[35] Bernabeu-Mora R, García-Guillamón G, Valera-Novella E, Giménez-Giménez LM, Escolar-Reina P, Medina-Mirapeix F. Frailty is a predictive factor of readmission within 90 days of hospitalization for acute exacerbations of chronic obstructive pulmonary disease: a longitudinal study. Ther Adv Respir Dis. 2017;11:383–92. 10.1177/1753465817726314 28849736 PMC5933665

[36] Garcia-Aymerich J, Farrero E, Félez MA, Izquierdo J, Marrades RM, Antó JM, et al Risk factors of readmission to hospital for a COPD exacerbation: a prospective study. Thorax. 2003;58:100–5 10.1136/thorax.58.2.100 12554887 PMC1746561

[37] Sokoreli I, Cleland JG, Pauws SC, Steyerberg EW, de Vries JJG, Riistama JM, et al Added value of frailty and social support in predicting risk of 30-day unplanned re-admission or death for patients with heart failure: An analysis from OPERA-HF. Int J Cardiol. 2019;278:167–172. 10.1016/j.ijcard.2018.12.030 30587417

[38] Braido F, Baiardini I, Menoni S, Bagnasco AM, Balbi F, Bocchibianchi S, et al Disability in COPD and its relationship to clinical and patient-reported outcomes. Curr Med Res Opin. 2011;27:981–6 10.1185/03007995.2011.563285 21385019

[39] Zapatero A, Barba R, Ruiz J, Losa JE, Plaza S, Canora J, et al Malnutrition and obesity: influence in mortality and readmissions in chronic obstructive pulmonary disease patients. J Hum Nutr Diet 2013;26:16–22. 10.1111/jhn.12088 23656492

[40] Lambert AA, Putcha N, Drummond MB, Boriek AM, Hanania NA, Kim V, et al COPDGene Investigators. Obesity Is Associated with Increased Morbidity in Moderate to Severe COPD. Chest. 2017;151:68–77. 10.1016/j.chest.2016.08.1432 27568229 PMC5310126

[41] Vitacca M, Paneroni M, Baiardi P, De Carolis V, Zampogna E, Belli S, et al Development of a Barthel Index based on dyspnea for patients with respiratory diseases. Int J Chron Obstruct Pulmon Dis. 2016;11:1199–206. 10.2147/COPD.S104376 27354778 PMC4907483

